# Comparison of morbidity at the donor site and clinical efficacy at the recipient site between two different connective tissue graft harvesting techniques from the palate: A randomized clinical trial

**DOI:** 10.34172/japid.2023.016

**Published:** 2023-09-12

**Authors:** Amine Beymouri, Siamak Yaghobee, Afshin Khorsand, Yaser Safi

**Affiliations:** ^1^Department of Periodontics, School of Dentistry, Tehran University of Medical Sciences, Tehran, Iran; ^2^Department of Oral and Maxillofacial Radiology, School of Dentistry, Shahid Beheshti University of Medical Sciences, Tehran, Iran

**Keywords:** Clinical efficacy, De-epithelialized gingival graft, Split-mouth, Subepithelial connective tissue graft, Trap door, Ultrasonography

## Abstract

**Background.:**

This study was conducted to compare the pain levels in patients and the clinical efficacy of grafts obtained using two techniques, namely de-epithelialized gingival graft (DGG) and subepithelial connective tissue graft (SCTG), in combination with coronally advanced flap (CAF) for the treatment of multiple adjacent gingival recessions.

**Methods.:**

Twelve patients were treated using DGG+CAF on one side and SCTG+CAF on the other. The patients’ pain levels at the surgical site, the number of analgesics taken on days 3 and 7, the mean root coverage (MRC), the percentage of complete root coverage (CRC), color match, and gingival thickness (GT) at the graft recipient site were evaluated 6 months after surgery.

**Results.:**

The total number of analgesics taken during the 7-day period after surgery and pain levels at the surgical site from day 3 to day 7 were significantly higher in the DGG+CAF group compared to the SCTG+CAF group (*P*=0.001). In the 6-month follow-up, color match and CRC were significantly higher in the SCTG+CAF group, while GT was significantly higher in the DGG+CAF group. There was no significant difference in MRC between the two groups.

**Conclusion.:**

The pain and analgesic consumption levels were higher in the DGG+CAF group compared to the SCTG+CAF group, and the recipient site had a weaker color match. However, this technique can lead to a greater increase in the thickness of the grafted area.

## Introduction

 Studies suggest that since subepithelial connective tissue graft (SCTG) provides better stability of the gingival margin and also creates some creeping attachment over time compared to other surgical methods, this technique, in combination with coronally advanced flap (CAF), is considered the gold standard for treating single or multiple gingival recessions,^1-3^ especially in cases where the gingiva is thin or there is minimal keratinized tissue around the tooth with recession.^4^ The most important complication associated with connective tissue grafts is increased patient morbidity at the donor site.^1,2,5^ Various techniques have been introduced for harvesting connective tissue grafts to achieve primary intention healing and reduce pain and bleeding at the donor site. However, implementing these techniques requires a sufficient palate thickness to prevent necrosis of the superficial flap.^6-8^ The most common technique is the trap-door technique^7^ described by Edel in 1974.^9^ Another technique is the single incision approach, initially introduced by Hürzeler and Weng^10^ in 1999. Lorenzana and Allen in 2000^11^ and Reino et al in 2013^12^ suggested modifications for harvesting connective tissue grafts using this method. These techniques are subcategories of SCTG. Another technique is the de-epithelialized gingival graft (DGG), which involves harvesting the graft in an epithelialized form and then de-epithelializing it. This method is easier and can be performed in areas with limited palatal tissue thickness.^7^ Moreover, it also causes less damage to the deep vascular and nerve structures of the palate.^13^ According to previous studies, in the process of obtaining a free gingival graft, secondary wound healing occurs, and complete healing takes place within 2‒4 weeks, depending on the thickness and width of the graft. In this approach, most patients experience pain and discomfort after the trauma, accompanied by occasional bleeding and delayed healing.^6,14,15^

 However, some recent studies comparing DGG and SCTG have shown no significant differences in bleeding, pain, discomfort, and analgesic consumption.^7,16^ These studies were conducted as parallel randomized clinical trials. Considering the variability in pain perception thresholds in different individuals, which can impact the results,^8^ it appears that a split-mouth design would be more appropriate for conducting such studies. Additionally, regarding clinical efficacy, some studies concluded that the graft obtained using the DGG technique was firmer and capable of creating a greater increase in gingival thickness (GT) compared to the SCTG technique.^3,7^ In contrast, a study did not confirm these findings.^16^ However, gingival piercing was used to measure the keratinized tissue thickness (GT) in these studies; this method increases the likelihood of errors, possibly due to the displacement of rubber stops, the file entering at different angles, the bending of fine files, and the stopping of thick files before reaching the bone level. Moreover, it requires anesthesia and poses a risk of infection.^17,18^ According to one study, the ultrasound method is a valid and repeatable technique for imaging periodontal tissues and is superior to the transgingival probing, which is an invasive method.^19^ Therefore, considering the existing controversies, a split-mouth design was used in the present study, and the resultant keratinized tissue thickness was measured using ultrasound.

 The primary objective of this study was to investigate the pain levels at the donor sites of the connective tissue graft and the amount of analgesics taken in two different techniques, namely SCTG + CAF and DGG + CAF. Additionally, the secondary objectives included assessing changes in keratinized tissue thickness, root coverage, color match, and graft shrinkage at the recipient sites in 6 months of follow-up.

## Methods

 This study was conducted on patients visiting the Periodontology Department of the School of Dentistry, Tehran University of Medical Sciences, from March 2022 to January 2023. Ten females and six males were included in the study. The study protocol was approved by the Ethics Committee of TUMS (ethical code: IR.TUMS.DENTISTRY.REC.1400.197), and the research was conducted in compliance with the Helsinki Declaration of 1975, as revised in 2013. The study findings were reported according to the 2010 CONSORT guidelines.

 This study was also registered in the Iranian Registry of Clinical Trials (identifier: IRCT20230102057017N1). All the patients provided informed consent before participating in the study.

###  Criteria for inclusion and exclusion in the study

- Presence of bilateral areas with teeth affected by gingival recession. If the recession areas were not similar in length on both sides, the area with the smaller extent was considered as the reference, and a surgical site with the same length was considered on the other side.
- Presence of bilateral recession areas with approximately similar recession depths.
- Lack of soft tissue interdental clinical attachment loss (CAL) in the recession areas (Miller Class 1 or 2).
- Presence of at least one tooth with a minimum recession depth of 2 mm on each side with recession.
- Lack of inflammation signs in the teeth located in the keratinized gingiva area (such as bleeding on probing, gingival enlargement, or suppuration). Periodontal treatment was conducted to control these signs if present.
- Presence of a sulcus with a depth of < 3 mm.
- Age over 18.
- No history of previous connective tissue graft harvesting from the palate.

Smokers, pregnant women, those taking medications that may affect periodontal tissues and their healing, and individuals with teeth in the surgical area that were mobile or had inadequate root canal treatment were excluded from the study.

###  Study design

 A split-mouth, randomized, controlled clinical trial with a parallel design was conducted on patients with bilateral gingival recession to compare two different connective tissue graft harvesting techniques.

 In this study, gingival grafts were harvested from the participants’ palates using the SCTG and DGG methods. The SCTG technique was considered the control group, and the DGG technique was considered the test group. Both the donor and recipient sites were located on the same side. Follow-up assessments were performed on days 3 and 7 and 6 months postoperatively. The final follow-up was conducted at six months, as previous studies have indicated that the maximum amount of shrinkage and dimensional changes in soft tissue grafts occur within the first six months after surgery.^5,20^

###  Sample size

 Based on the results of a study by Zucchelli et al^7^ in 2010 and using the Paired Means Power Analysis option in PASS 11 software, with α = 0.05, β = 0.2, and anticipating approximately 25% loss of follow-up, a sample size of 16 individuals was considered.

###  Randomization

 Four envelopes were labeled as SCTG-right, SCTG-left, DGG-right, and DGG-left to determine the side and type of surgery. On the day of surgery and immediately before starting the procedure, one of the envelopes was opened randomly to determine the side and type of surgery for the first session. Approximately 2‒4 weeks later (depending on healing), the other surgical method was performed on the opposite side.

###  Measurements

 One examiner performed all the clinical and ultrasonic measurements. An intra-examiner calibration was conducted before starting the study to reduce the possibility of error. According to the ICC test, a value of 0.9 confirms the reliability of the measurements. In this study, the ICC value was 0.989, indicating the reliability of the measurements.

###  Preoperative Measurements

####  Full-mouth plaque score 

 In the first session, the patients underwent scaling, root planing, and oral hygiene instructions. One week later, the patients were re-examined, and if their full-mouth plaque score (FMPS), according to the O’Leary index,^21^ was < 20%, a surgery appointment was scheduled for them.

####  Palatal thickness

 For this purpose, after administering local anesthesia and before starting the surgery, the thickness of the palatal mucosa was measured by probing to the bone in the second premolar area. The Williams periodontal probe (D&P) was used for all measurements and recorded to the nearest 0.5 mm.

####  Recession depth

 The distance from the cementoenamel junction (CEJ) to the gingival margin at the most apical part was measured using a probe for each tooth.

###  Intraoperative measurements

####  Graft thickness

 Since the thickness of the harvested graft from the palate should be approximately the same in both groups, after removing the adipose and glandular tissues in the SCTG group and removing the epithelium and adipose tissue in the DGG group, the thickness was measured at two points using a graduated gauge. These two points were located 2 mm from the mesial and distal edges and at the center of the graft height. Then, the two measurements were averaged as the graft thickness.

####  Graft area

 Before suturing, a photograph was taken of the harvested graft. Then, the baseline area of the graft was measured using the ImageJ software.

####  Covering flap thickness

 The flap thickness was measured at the center at a distance of 2 mm from the margin using a gauge to ensure that the thickness of the flap was approximately the same on both sides.

###  Postoperative measurements

####  Pain level

 To assess the pain level at the donor site, the patient was asked to rate their pain on days 3 and 7 using the numeric analog scale (NAS), ranging from 0 to 10, with 0 representing no pain and 10 representing the worst possible pain. Additionally, the number of analgesics taken by the patient was recorded. On day 3, the patients were contacted by phone to inquire about their pain score and the number of analgesics taken. On day 7, during an in-person visit, the patients were asked to report their pain scores and the number of analgesics taken from day 3 to 7.

####  Color matching

 To evaluate the color matching after 6 months, three blinded professors were asked to rate the color matching of the grafted areas with the surrounding tissues on a scale of 0 to 10. Then, the average score for each side was calculated, and the side with the higher score was considered to have better color matching.

####  Graft shrinkage

**Figure 1:**
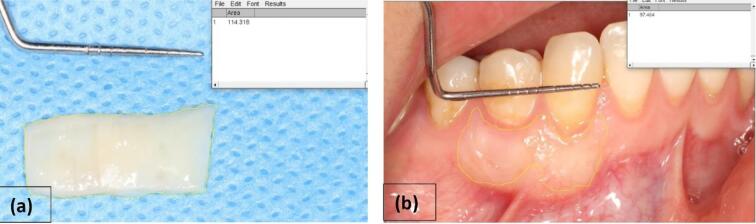
Calculation of graft shrinkage by the ImageJ software: (a) calculating the graft area before suturing, (b) calculating the grafted area 6 months after surgery

 In the follow-up visit after 6 months, photographs of the grafted area were taken, and the area was measured using the ImageJ software. The difference between the baseline area and the area after 6 months was calculated to determine the amount of shrinkage (Figure 1).

###  Mean root coverage 

 Mean root coverage (MRC) was calculated using the below formula:


$$\frac{baseline\;RD-6\;months\;RD}{baseline\;RD}\times 100\%$$

###  Complete root coverage 

 The number of defects treated in which the soft tissue margin was placed at the level of CEJ or more coronal than that was divided by the total number of defects. The resulting number was then multiplied by 100 and expressed as a percentage.

###  Keratinized gingival thickness

**Figure 2:**
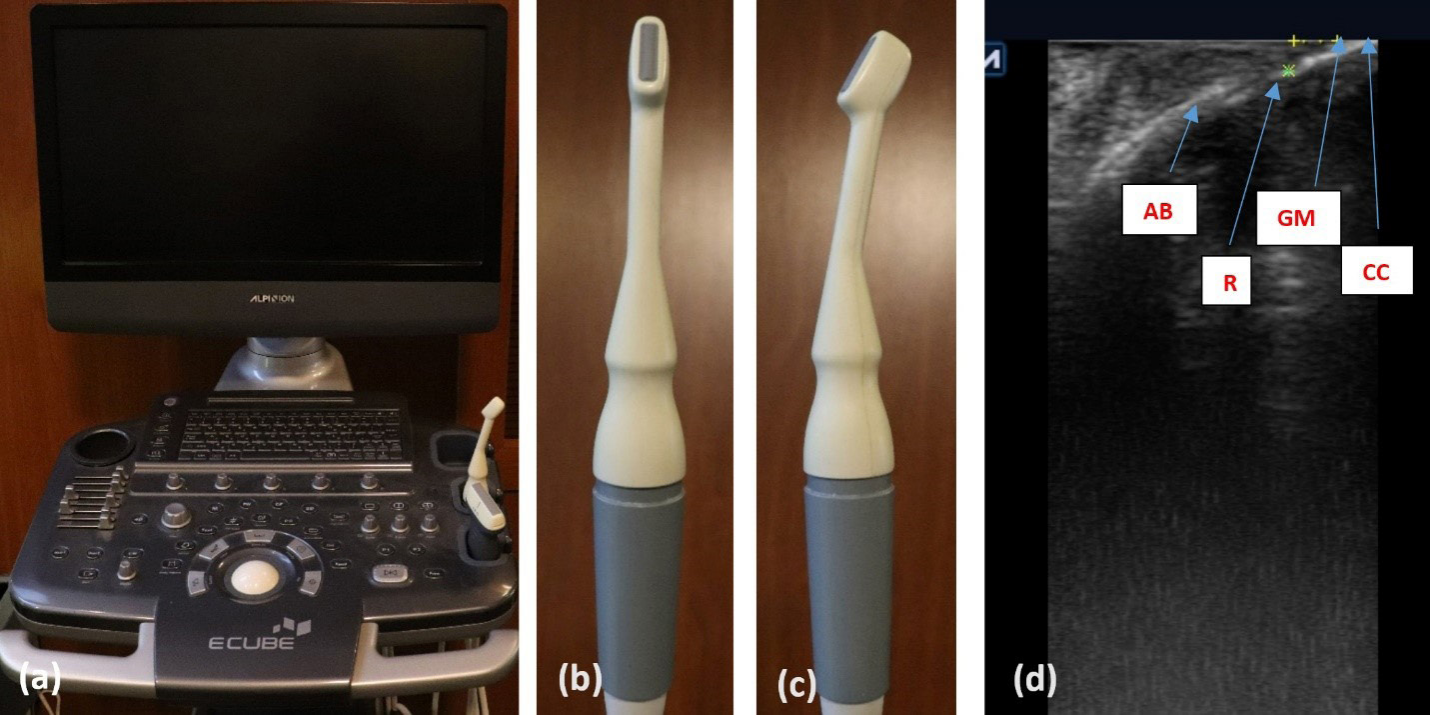
Ultrasound measurement: (a) ultrasound device (ECUBE 7, ALPINION Company) used in this study; (b) intraoral probe for gingival thickness measurement in front view; (c) lateral view of intraoral probe; (d) anatomic areas on ultrasonography; gingival thickness measurement on the root, 2 mm apical from the gingival margin. Note that this case has CRC and gingival margin adopted to CEJ. CC: clinical crown, GM: gingival margin, R: root, AB: alveolar bone

 Six months after the surgery, in the teeth with recession depth > 2 mm, measurements were taken at a distance of 2 mm from the CEJ in the mid-buccal region using an ultrasound device (ECUBE 7, ALPINION Company) equipped with a probe with a miniature-sized linear array (IO3-12) for intraoral measurements. In this method, a transducer array generates acoustic waves, and an image is formed by analyzing the reflected waves.^22^ In these images, structures such as the enamel appear as hyperechoic (bright) regions, while soft tissues appear as hypoechoic (dark) areas. Therefore, it is almost impossible to distinguish the exact location of the CEJ where the hyperechoic structures of enamel and cementum meet. Consequently, the gingival margin location determined clinically was used. In this study, except for two patients, all others had at least one tooth with complete root coverage (CRC) on each side of the gingival margin’s location, indicating the CEJ’s location and GT measurements were made on those teeth. In the two mentioned patients, teeth with a remaining RD of 0.5 mm were present on each side. Therefore, in the ultrasonography image, the thickness of GT was determined to be 1.5 mm apical to the gingival margin (2 mm from the CEJ) (Figure 2).

###  Surgical procedure

 One surgeon performed all surgeries. Before the surgery, the patients were instructed to use a mouthwash of 0.12% chlorhexidine for one minute. After administering local anesthesia with 2% lidocaine with epinephrine (Persocaine-E, Darou Pakhsh Co, Iran), the recipient site for the graft was prepared.

 In the flap design, horizontal incisions were made sub-marginally in the interdental areas, which extended to the sulcular incisions on the buccal side. The distance of these incisions from the tip of the anatomical papilla was equal to the recession depth plus 1 mm. The flap extension involved one or more teeth on the mesial and distal sides affected by the recession, and the coronal portion of the horizontal incision was de-epithelialized at the anatomical papilla to provide a bed for surgical papilla.^23^ The flap was placed coronally to the CEJ without tension.

 To obtain a graft from the donor site, a template was first prepared. In this study, all the grafts were obtained from the mesial of the first premolar to the distal of the second molar, based on the quality and thickness of the keratinized tissue.

**Figure 3:**
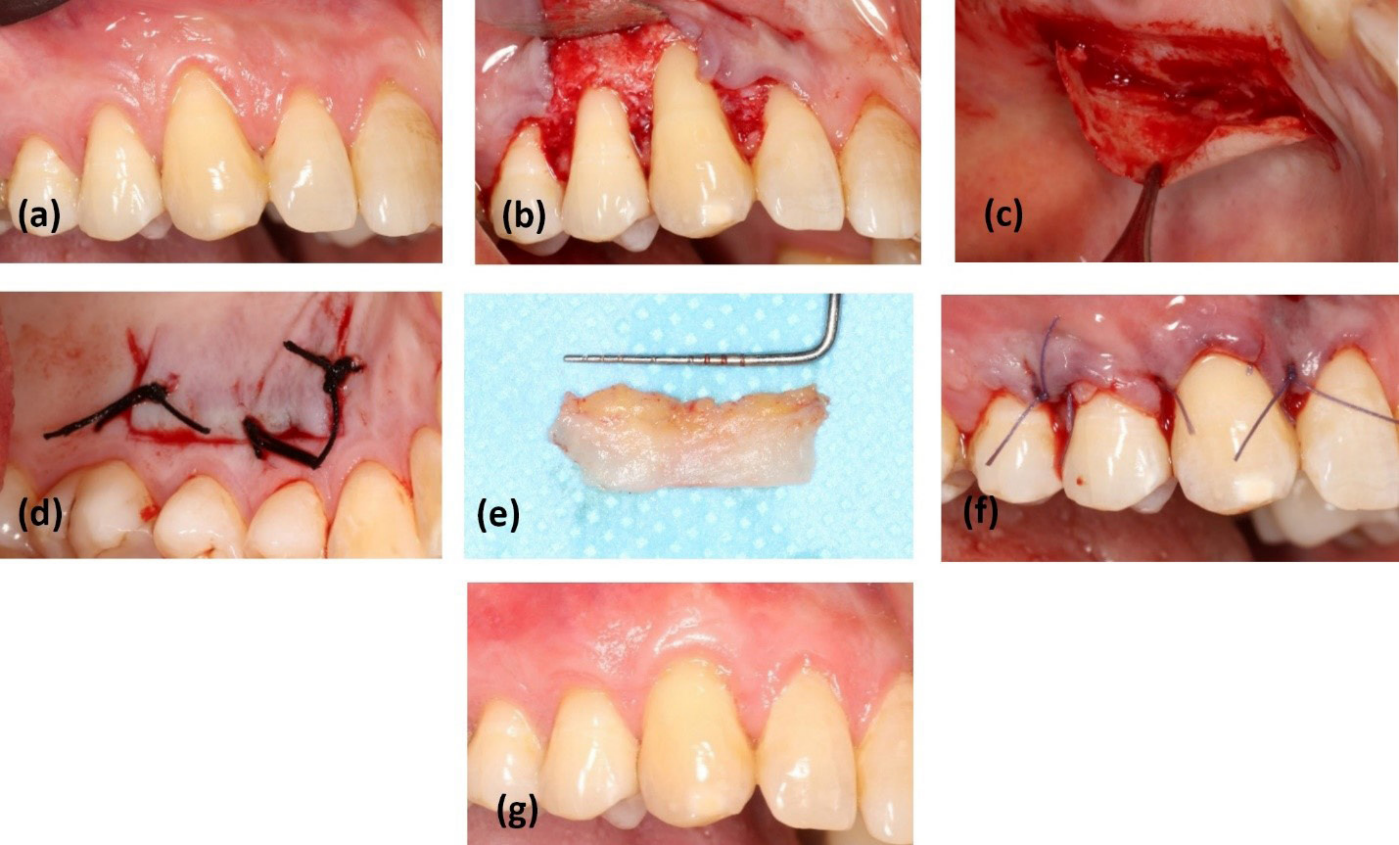
Surgery in SCTG + CAF: (a) baseline; (b) bed preparation at the recipient site; (c) split flap elevated in palate to harvest SCTG by the Trap-door technique; (d) suturing the window; (e) SCTG after harvesting; (f) suturing the graft on denuded roots and flap in the coronal position; (g) healing after 6 months of follow-up. SCTG: sub-epithelial connective tissue graft, CAF: coronally advanced flap

 In the control group, the trap-door technique^9^ was used as SCTG. The initial flap was raised as a split-thickness flap to create access to the graft through the created widow. Care was taken not to disturb the periosteum. After obtaining the graft, the adipose tissue was removed from the inner surface. For suturing the graft, 3-0 silk sutures (SUPASIL, Iran) and the interrupted technique were used (Figure 3).

**Figure 4:**
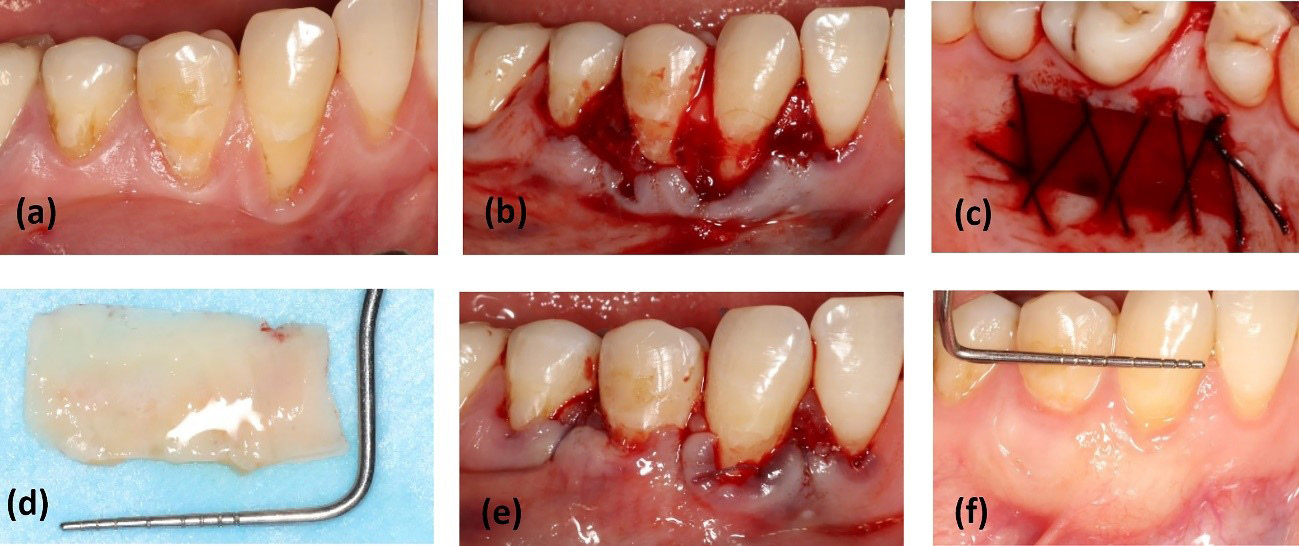
Surgery in DGG + CAF: (a) baseline; (b) bed preparation at the recipient site; (c) harvesting a gingival graft from the palate and suturing the palate by the cross mattress technique; (d) de-epithelialization of the gingival graft; (e) suturing the graft on denuded roots and flap in the coronal position; (f) healing after 6 months of follow-up. DGG: de-epithelialized gingival graft, CAF: coronally advanced flap

 The gingival graft, consisting of superficial epithelium and subepithelial connective tissue, was separated from the palate in the test group. Outside the mouth, under direct light, the epithelial, adipose, and glandular tissues were removed from the graft using a #15 surgical blade (Paramount Surgimed Ltd, India).^7^ For suturing the palate, 3-0 silk (SUPASIL, Iran) and the cross mattress technique were used (Figure 4).

 After root planing using Gracey curettes (Hu-Friedy, USA), the graft was fixed in the recipient site by interrupted sutures using resorbable polyglycolic acid 4-0 (Tajhiz Gostar Tamin Salamat Co, Iran). Subsequently, the graft was compressed with moist gauze for 1 minute to achieve better adaptation with the recipient site. Finally, the superficial flap was sutured 1-2 mm coronally to the CEJ,^1^ and a periodontal pack (GC America Inc, USA) was placed over the donor area.

###  Postsurgical protocol

 The patients were asked to immediately take a 400-mg ibuprofen (ADVIFEN 400) tablet after the surgery. Then, they were instructed to take a second dose 6 hours later and use subsequent doses as needed. The patients were educated not to brush their teeth in the surgical area for 3 weeks and to use 0.12% chlorhexidine mouthwash twice daily for 1 minute. Additionally, they were prescribed 500-mg amoxicillin (Amoxicillin, Kosar Co, Iran) capsules every 8 hours for 1 week.

 One week after the surgery, the periodontal pack and sutures at the donor site were removed. The sutures at the recipient site were also removed two weeks after surgery. Three weeks postoperatively, the patients were trained to use a soft toothbrush to brush their teeth with the roll technique for one month. The patients were visited for check-ups throughout the study and received oral hygiene instructions every two months.

###  Statistical analysis

 In this study, the variables were compared between the two groups and within one group at different time intervals using the paired t-test. A *P* value ≤ 0.05 was defined as statistically significant. Additionally, the McNemar test was used to calculate the CRC in both groups.

## Results

**Figure 5:**
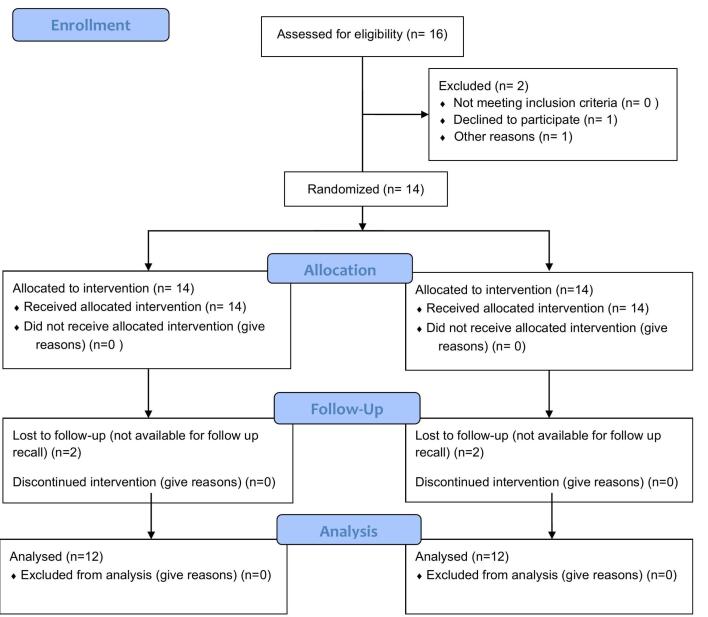
The CONSORT flow chart

 Of 16 patients, 12 (8 females and 4 males), with a mean age of 46 years (ranging from 19 to 61 years), underwent surgical treatment and completed the 6-month follow-up. Figure 5 shows the CONSORT flow chart regarding the number of patients. In total, 52 teeth affected by recession were treated without any specific complications. Table 1 shows the characteristics of the teeth and the mean thickness of the graft, flap, and palate in both groups.

**Table 1:** Characteristics of involved teeth, the thickness of grafts, flaps, and palatal tissues

**Variables**	**DGG+CAF (n=27 teeth)**	**SCTG+CAF (n=25 teeth)**
Tooth location		
maxilla	7	12
Mandible	20	13
Type of teeth		
Upper incisors	2	3
Lower incisors	0	0
Upper canine	3	4
Lower canine	7	5
Upper premolars	2	4
Lower premolars	11	8
Upper molars	0	1
Upper molars	2	0
Palatal thickness	3.31 ± 0.25	3.18 ± 0.25
Graft thickness	1.11 ± 0.11	1.07 ± 0.11
Flap thickness	0.26 ± 0.06	0.25 ± 0.05

###  Postoperative pain outcomes

 In terms of pain level and analgesic consumption up to the third day, the DGG + CAF group experienced higher pain and analgesic use, but this difference did not reach statistical significance. However, from the third to the seventh day, pain intensity and the number of analgesics consumed were significantly higher in the DGG + CAF group. Moreover, the total number of analgesics used was significantly higher in the DGG + CAF group compared to the SCTG + CAF group (Table 2).

**Table 2:** Postoperative pain

**Pain parameters**	**DGG+CAF**	**SCTG+CAF**	***P***** value**
Postoperative pain			
3 days	2.42 ± 1.44	1.67 ± 1.07	0.069
7 days	2.33 ± 2.18	0.42 ± 0.51	0.009*
*P*value	0.183	0.154	
Amount of analgesic intake (in number)			
3 days	7.67 ± 3.75	4.75 ± 2.98	0.058
7 days	5.58 ± 4.60	1.83 ± 3.32	0.015*
*P* value	0.628	0.043*	
Total number of analgesics	13.25 ± 5.46	6.58 ± 5.63	0.001*

*Significant at *P* ≤ 0.05.

###  Clinical parameters

 There was no statistically significant difference in MRC between the two groups. The MRC was 87.18 ± 13.46% and 88.28 ± 9.86% in the DGG + CAF and SCTG + CAF groups, respectively. However, regarding CRC, the SCTG + CAF group showed significantly better results (64% versus 44%).

 As for GT during the 6-month follow-up, the DGG + CAF group showed significantly better results with a thickness of 1.36 mm compared to 1.1 mm in the SCTG + CAF group. The amount of shrinkage was 33.91 ± 15.05% and 43.5 ± 9.12% in the DGG + CAF and SCTG + CAF groups, respectively. Despite the lower percentage of shrinkage in the de-epithelialized group, this difference was not statistically significant. Regarding color matching after 6 months, the SCTG group demonstrated significantly better color matching, with a mean score of 9.3, compared to the DGG group, with a score of 7. Table 3 displays the clinical parameters of the study at baseline and 6 months postoperatively.

**Table 3:** Clinical parameters at baseline and 6 months follow up

**Clinical parameters**	**DGG+CAF**	**SCTG+CAF**	***P***** value**
RD (mm)			
Baseline	2.72 ± 0.65	2.89 ± 0.75	0.371
6 months	0.41 ± 0.28	0.40 ± 0.31	0.864
*P *value	0.001*	0.001*	
GT (mm)			
Baseline	0	0	
6 months	1.36 ± 0.23	1.10 ± 0.17	0.001*
*P* value	0.001*	0.001*	
Shrinkage (%)			
6 months	33.91 ± 15.05%	43.5 ± 9.12%	0.082
Color matching			
6 months	7.00 ± 0.79	9.33 ± 0.44	0.001*
MRC (%)			
6 months	87.18 ± 13.46%	88.28 ± 9.86%	0.345
CRC (%)			
6 months	46%	64%	

Note: RD: recession depth, GT: gingival thickness, MRC: mean root coverage, CRC: complete root coverage. * Significant at *P* ≤ 0.05.

## Discussion

 This study aimed to compare pain during the harvesting of connective tissue grafts using two different techniques, namely SCTG and DGG, from the palate and also investigate the clinical efficacy of the grafts obtained with these two methods in combination with CAF. To the best of the authors’ knowledge, this study is the first study on multiple gingival recessions with a split-mouth design using ultrasonography to measure GT.

 The thickness of the grafts obtained in the patients of this study ranged from 1.3 to 0.9 mm. Previous studies have considered a thickness of 1 mm suitable for obtaining connective tissue grafts.^13^ There was no significant difference in the thickness of the grafts taken from two sites in the same patient.

 According to the results of this study, the level of pain at the donor site and the consumption of analgesics from the third day to the seventh day were significantly higher in the DGG group, indicating that pain decreased significantly until the third day in the SCTG group, while it persisted in the DGG group. Furthermore, the patients in the DGG group consumed a significantly higher number of analgesics for pain control from the day of surgery to seven days postoperatively (13.25 ± 5.46 versus 6.58 ± 5.63), consistent with the findings of studies by Wessel and Tatakis^8^ and Del Pizzo et al.^6^ Mashaly et al^3^ also concluded that the level of chewing disability, stress, and pain on the third day was higher in the DGG method compared to the SCTG method. However, Zucchelli et al^7^ found no significant difference in the pain level and analgesic consumption between the two techniques. In their study, 28% of the individuals in the trap-door group experienced dehiscence or necrosis of the superficial flap, which increased the pain score. In the present study, all the patients had a minimum palatal mucosa thickness of 3 mm, and no cases of superficial flap necrosis were observed in the trap-door group, possibly because, according to previous studies, the risk of superficial flap necrosis is higher if the palatal mucosa thickness is less than 2.5 mm.^24,25^ Furthermore, the study by Zucchelli et al^7^ focused on single recession sites. In smaller grafts, the difference in pain between the two techniques may not be very noticeable. Bakhishov et al^16^ also found no significant differences in pain, discomfort, and analgesic consumption between DGG and SCTG groups. However, it is worth noting that in that study, SCTG was obtained using the single incision technique, where the connective tissue graft is harvested with periosteum. This difference in technique could potentially justify variations in the study results.

 Bertl et al^26^ conducted a study on cadavers and found that an increase in palatal mucosa thickness did not lead to an increase in the lamina propria thickness; instead, the submucosal thickness increased. On average, the lamina propria thickness ranges between 1.5 and 2 mm in the marginal area and between 0.9 and 1.4 mm in the apical area of the palate. Therefore, in the SCTG method, regardless of the palatal mucosa thickness, it is always expected to have a significant portion of glandular and fatty tissue. This tissue is prone to significant shrinkage, resulting in increased graft shrinkage.^3,26^ In contrast, in the DGG method, due to the presence of more collagen fibers in the lamina propria, the graft is denser and undergoes less shrinkage.^13,16,27^ This study confirmed the findings of previous studies, although the difference between the two groups in terms of graft shrinkage did not reach statistical significance at the 6-month follow-up.

 One of the secondary objectives of this study was to compare the level of root coverage. The MRC was 88.28% ± 9.86% and 87.18% ± 13.46% in the SCTG and DGG groups, respectively, indicating no significant difference between the two groups, consistent with the findings of Zucchelli et al^7^ and Mashaly et al.^3^ However, a systematic review by Tavelli et al^28^ in 2019 indicated that the MRC was 94% in the DGG method and 89.3% in the SCTG method, favoring the DGG technique.

 The obtained CRC value was 64% and 44% in the SCTG and DGG groups, respectively, indicating that the SCTG group was more successful in achieving CRC. However, this superiority has not been demonstrated in similar studies.^3,7,16^ The CRC value for the DGG group in our study was also lower compared to the findings of Zucchelli et al,^7^ Mashaly et al,^3^ and Bakhishov et al,^16^ who reported CRC values of 84%, 93%, and 70%, respectively. It is worth noting that the studies by Zucchelli and Bakhishov assessed CRC at 12 months postoperatively. Considering the possibility of creeping attachment,^1^ an increase in CRC over time is plausible.

 The percentage of CRC for the SCTG group in this study was lower than the percentages reported by Zucchelli et al^7^ and Mashaly et al,^3^ which were 72% and 93%, respectively, and higher than the percentage reported by Bakhishov et al,^16^ which was 48%. This variation in the CRC percentage could be attributed to different methodologies and various SCTG harvesting techniques used in these studies.

 In general, the results of root coverage are influenced by three groups of factors: patient-related factors, including smoking among other factors; site-related factors, including aspects such as Miller’s classification, recession depth and width, GT, and the amount of keratinized tissue in the apical part of the recession area, vestibular depth, presence of high frenum attachments, and previous restorations in the recession area; and technique-related factors consisting of gingival margin placement relative to CEJ, flap tension, the use of vertical releasing incisions, and the adoption of microsurgical techniques.^2,6,29^ Matching all these factors for patients and even within an individual’s quadrants is not easy. These factors can affect root coverage outcomes.

 Gingival grafts that are de-epithelialized outside the oral cavity have a high probability of scar formation in the recipient area, leading to weaker color and texture matching of these grafts. In fact, the behavior of these grafts becomes more similar to free gingival grafts, which might be due to the presence of islands of epithelium in the graft, especially in areas where the epithelium interlocks with the lamina propria.^13^ Many studies have confirmed the presence of epithelial remnants after de-epithelialization of gingival grafts in histological investigations.^16,30-32^ Another possible reason may be the high inducibility of the epithelial cell differentiation in the recipient tissue by the superficial layers of the connective tissue.^33^ In this study, the DGG + CAF technique exhibited a weaker color match with an average score of 7 compared to SCTG + CAF, with an average score of 9.3.

 The obtained GT value was 0.25 mm higher in the DGG + CAF group compared to the SCTG + CAF group, indicating a statistically significant difference. Consistent with this study, Mashaly et al^3^ and Zucchelli et al^7^ also found that the DGG method showed a greater increase in GT. The studies attributed this to the denser and more stable connective tissue closer to the epithelium in the DGG method,^7^ which is less compressed by the CAF pressure.^26^

 One of the limitations of this study was the difficulty in determining the boundaries of the grafted area after 6 months, especially in the SCTG technique due to its high color match. The images were examined with various magnifications, but there is still a possibility of error in measuring the amount of shrinkage. Additionally, there was a 25% drop-out in patients.

 This study suggests further research on the possibility of recession depth relapse in these two techniques, requiring follow-ups of at least 5 years and larger sample sizes.

## Conclusion

 With all the limitations considered, this study concluded that the patients in the DGG + CAF group experienced higher pain and morbidity levels than the SCTG + CAF group, and the harvested area showed weaker color match. However, this technique can potentially achieve a greater increase in the thickness of the harvested area, which may be effective in reducing the risk of relapse in treated regions.

## Acknowledgments

 This study was scientifically and technologically supported by the Tehran University of Medical Sciences, and special thanks to Dr. Yaser Safi’s dental radiology center, where ultrasonography measurements were made.

## Competing Interests

 The authors declare that they have no competing interests.

## Data Availability Statement

 The data from the reported study are available upon request from the corresponding author.

## Ethical Approval

 The study protocol was approved by the Ethics Committee of Tehran University of Medical Sciences (ethical code: IR.TUMS.DENTISTRY.REC.1400.197).

## Funding

 None.
